# Transcriptome analysis indicated that *Salmonella* lipopolysaccharide-induced thymocyte death and thymic atrophy were related to TLR4-FOS/JUN pathway in chicks

**DOI:** 10.1186/s12864-016-2674-6

**Published:** 2016-05-04

**Authors:** Haibo Huang, An Liu, Hui Wu, Abdur Rahman Ansari, Jixiang Wang, Xiyao Huang, Xing Zhao, Kemei Peng, Juming Zhong, Huazhen Liu

**Affiliations:** Department of Basic Veterinary Medicine, College of Animal Science and Veterinary Medicine, Huazhong Agricultural University, Wuhan, 430070 China; Key Lab of Animal Genetics, Breeding and Reproduction of Ministry Education, College of Animal Science and Technology, Huazhong Agricultural University, Wuhan, 430070 China; Department of Anatomy, Physiology and Pharmacology, College of Veterinary Medicine, Auburn University, Auburn, AL 36849 USA

**Keywords:** *Salmonella typhimurium*, Thymus injury, chT1, Transcriptome

## Abstract

**Background:**

Thymus is the crucial site for T cell development and once believed to be immune privileged. Recently, thymus has gained special attention as it is commonly targeted by infectious agents which may cause pathogenic tolerance and subsequent immunosuppression.

**Results:**

We analyzed thymic responses to the challenge with *Salmonella typhimurium* (STm) or lipopolysaccharide (LPS) derived from STm in chicks. Newly hatched chicks were injected intraperitoneally with 5 × 10^4^ CFU/mL STm or 50 mg/kg LPS. After LPS treatment, maximum thymocyte death (3 ~ 5-fold change) compared to controls was found at 12 h, and maximum loss of thymic weight (35 %) and reduced thymic index (20 %) were found at 36 h. After STm infection, maximum thymocyte death and thymic atrophy occurred at 36 and 72 h, respectively. No significant changes of thymic structure, chT1+ and CD4+/CD8+ T cell ratio were observed in thymus or spleen tissues after LPS treatment. Furthermore, transcriptome analysis revealed important roles for the TLR4-FOS/JUN signaling pathway in thymic injury. Thus, the major process of thymic atrophy in this study first involved activation of transcriptional factors FOS/JUN upon LPS binding to TLR4 that caused release of inflammatory factors, thereby inducing inflammatory responses and DNA damage and ultimately cell cycle arrest and thymic injury.

**Conclusions:**

STm and *Salmonella* LPS could induce acute chick thymic injury. LPS treatment acted faster than STm. TLR4-FOS/JUN pathway may play an important role in LPS induced chick thymic injury.

**Electronic supplementary material:**

The online version of this article (doi:10.1186/s12864-016-2674-6) contains supplementary material, which is available to authorized users.

## Background

The thymus is the primary immune organ providing naïve T cells for peripheral immune tissues [1]. Theoretically, thymic injury can cause serious consequences, which are related to local tissue homeostasis and immune development, especially in young individuals with an immature immune system. Due to the existence of the blood-thymus barrier, the thymus was once deemed to be immune privileged [2]. However, in mammals, multiple pathogens can target the thymus and cause thymic injury, including extensive cell death, tissue structure abnormality, organ atrophy and functional disorder, including recent thymic emigrant abnormality and T cell tolerance to pathogens [3, 4]. In birds, pathogens including viruses (e.g., chicken anemia virus, Marek’s disease virus and avian leukosis virus), bacteria (e.g., *Escherichia coli*) and parasites (e.g., *Ascaridia galli*) can also induce thymic atrophy [5–9]. Therefore, thymus injury also may be a common occurrence during infection in birds.

*Salmonella typhimurium* (STm) is one of the most deleterious food-borne pathogens that can induce typhoid fever and enteritis through infected chicken eggs and meat [10]. STm infection could also cause the death of newly hatched chicks [11]. STm infection has been reported to induce thymic atrophy, thymocyte death and a modest decrease of recent T cell export in mice [12, 13]. Moreover, one study indicated that the thymic injury induced by STm in mice was mediated by intracellular JNK signaling and its downstream effectors, including reactive oxygen species (ROS) and inflammatory cytokines (e.g., TNF-α and IFN-γ) [13]. In chickens, several studies have indicated that STm infection can induce expression of chemokines and cytokines (e.g., IL-8 and IL-1β) in the spleen, liver and intestinal tissues, which may contribute to the host defense against STm [14, 15]. However, unlike in mammals, few studies have reported the impact of STm infection on the thymus in birds, and the molecular mechanism of chicken thymus response to STm infection is largely unknown.

In the present study, we evaluated the thymic injury and potential mechanisms induced by both STm and *Salmonella* LPS in newly hatched chicks. We found that both STm and LPS stimulation could induce acute thymus injury in chicks. The effects of LPS are faster than that of STm. Moreover, we found that the TLR4-FOS/JUN signaling pathway may play a key role in this process. Our results offer novel evidence for the molecular mechanism of the thymus injury induced by *Salmonella* LPS.

## Results

### Thymic atrophy after LPS treatment

**Fig. 1:**
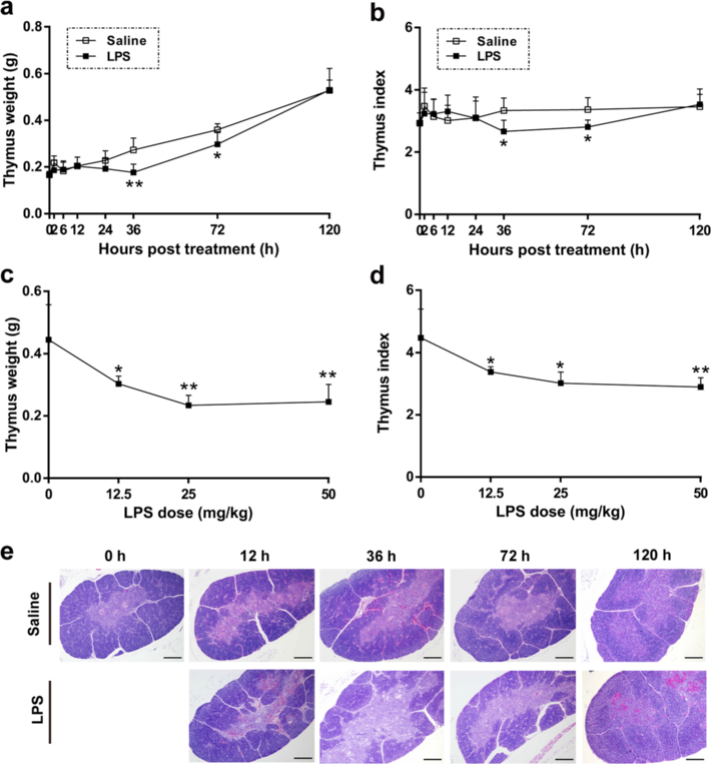
*Salmonella* LPS induced acute thymic atrophy in chicks. Newly hatched chicks were injected i.p. with saline or *Salmonella* LPS and then sacrificed at defined time points to analyze thymus weight and index. **a**, **b** LPS treatment (50 mg/kg) reduced thymus weight (**a**) and index (**b**) in chicks (*n* = 4 ~ 6) at 36 and 72 hpt. Statistically significant differences between LPS and saline groups at each time point were determined using Student’s *t*-test. (**c**, **d**) LPS treatment decreased chick thymic weight (**c**) and index (**d**) in a dose-dependent manner (*n* = 3 ~ 5) at 36 hpt. Statistically significant differences between multiple LPS dose groups versus control group (0 mg/kg LPS) was performed with Bonferroni’s multiple comparisons test after one-way ANOVA test. **e** Sections of thymuses from LPS (50 mg/kg) or saline treated chicks were stained with hematoxylin and eosin to analyze changes of tissue structure. Light areas represent the medulla, and dark areas represent the cortex. Scale bars = 200 μm. All data are presented as means ± SD. *P < 0.05, **P < 0.01

Newly hatched chicks were injected i.p. with 5 × 10^4^ CFU/mL STm or 75 % saline (control) for the challenge of STm, and with 50 mg/kg *Salmonella* LPS or 75 % saline (control) for the challenge of LPS. The thymic weight was assessed at 0 ~ 120 h post treatment (hpt). Compared with saline treatment, treatment with LPS derived from STm resulted in the maximum 35 % loss of thymus weight (*P* < 0.01, 0.18 ± 0.04 g vs. 0.27 ± 0.05 g) and 20 % decrease of thymus index (*P* < 0.05, 2.66 ± 0.37 vs. 3.34 ± 0.40) at 36 hpt; meanwhile, it caused 17 % loss of thymus weight (*P* < 0.05, 0.30 ± 0.05 g vs. 0.36 ± 0.03 g) and 17 % loss of thymus index (*P* < 0.05, 2.81 ± 0.23 vs. 3.37 ± 0.38) at 72 hpt (Fig. 1a and b). The thymus weight was restored to the normal level at 120 hpt (Fig. 1a and b). Moreover, there is a dosage effect of LPS on thymic weight (*P* < 0.05) and index (*P* < 0.05) (Fig. 1c and d). Compared with saline treatment, STm infection resulted in the maximum 43 % loss of thymus weight (*P* < 0.01, 0.13 ± 0.02 g vs. 0.22 ± 0.05 g) and 26 % decrease of thymus index (*P* < 0.05, 1.86 ± 0.25 vs. 2.52 ± 0.55) at 72 hpt compared with controls, and these effects were attenuated at 120 hpt (Additional file 1: Figure S1). Furthermore, the thymus maintained structural integrity with distinct boundaries of the medulla and cortex at different stages of LPS treatment (Fig. 1e). Therefore, both STm and *Salmonella* LPS treatment induced acute thymic atrophy, but the maximum LPS-induced thymic atrophy occurred 36 h earlier than that caused by STm infection.

### Thymocyte death after LPS treatment

**Fig. 2:**
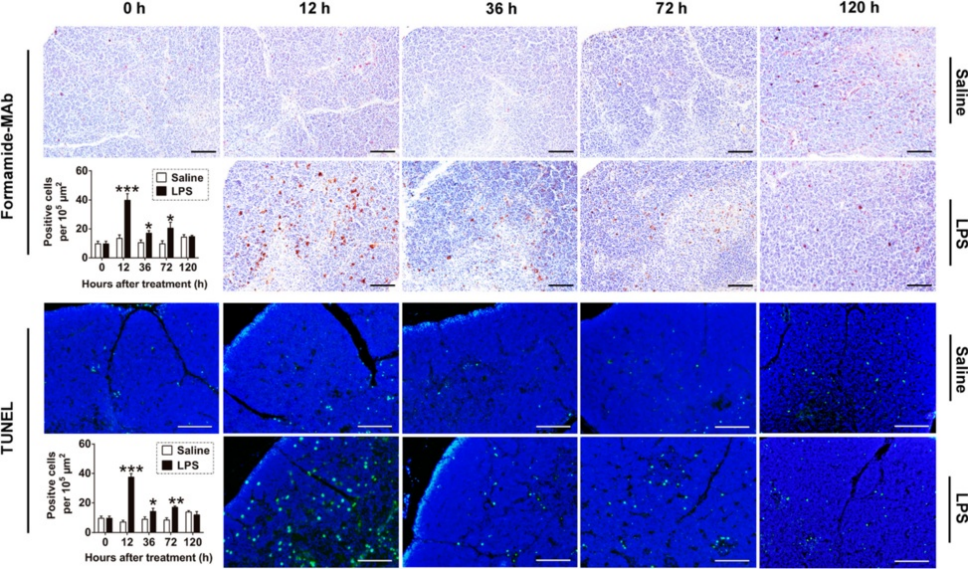
*Salmonella* LPS induced acute thymocyte death in chicks. Newly hatched chicks were injected i.p. with saline or 50 mg/kg *Salmonella* LPS and then sacrificed at defined time points (*n* = 3 ~ 6) to analyze cell death by the formamide-MAb assay and TUNEL assay. The positive cells were mainly distributed in thymic cortex. At least 5 fields in each section of the thymus were sampled, and positive cells per 1 × 10^5^ μm^2^ in the thymic cortex were quantified. Scale bars = 50 μm. Data are presented as means ± SD. Statistically significant differences between LPS and saline groups at each time point were determined using Student’s *t*-test. *P < 0.05, **P < 0.01, ***P < 0.001

We assessed thymocyte death using two classic methods, the formamide-MAb assay, which recognizes damaged single-strand DNA in early apoptotic cells [16–18], and the TUNEL assay, which detects low-molecular-weight damaged DNA fragments in late apoptotic cells or necrotic cells [17, 19]. These damaged DNA fragments are abundant in apoptotic cells [16–19]. The formamide-MAb assay revealed that the maximum LPS-induced thymocyte death occurred at 12 hpt, which was 3-fold higher than that of the control (*P* < 0.001, 40 ± 11.00 vs. 14 ± 5.65 per 10^5^ μm). Additionally, the positive cells were about 2-fold higher at 36 and 72 hpt. These effects have also been detected by the TUNEL assay. The results of TUNEL assay were almost identical to that of the formamide-MAb assay, and the TUNEL positive cells were about 5-fold higher than that of the control at 12 hpt (Fig. 2). During STm infection, the maximum rate of thymocyte death occurred at 36 h, and the positive cells were 4-fold higher than that of control (*P* < 0.01, 40 ± 15.96 vs. 11 ± 1.57 per 10^5^ μm). They were attenuated by 2-fold (*P* < 0.001, 19 ± 2.44 vs. 10 ± 1.07 per 10^5^ μm) at 72 hpt as detected by the formamide-MAb assay (Additional file 1: Figure S1). Therefore, both STm and *Salmonella* LPS treatment induced thymocyte death, and the maximum death of thymocytes occurred 24 ~ 36 h ahead of the peak organic atrophy. Moreover, LPS-induced thymocyte death occurred 24 h earlier than that stimulated by STm infection (Fig. 2; Additional file 1: Figure S1), which coincided with the phenomenon of LPS-mediated thymus atrophy ahead of that induced by STm infection (Fig. 1; Additional file 1: Figure S1). In all cases, thymocyte death was mainly located in the thymic cortex.

### Maintenance of chT1+ and CD4+/CD8+ cell ratios at 36 h after LPS treatment

**Fig. 3:**
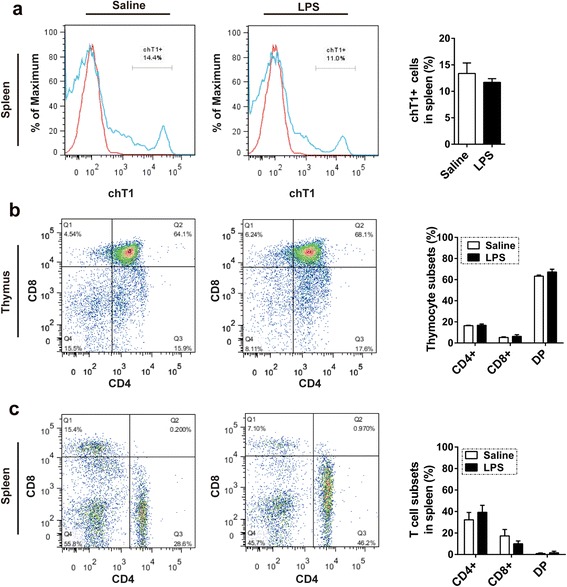
Impact of *Salmonella* LPS on T cell population in chick thymus and spleen. Newly hatched chicks were injected i.p. with saline or 50 mg/kg *Salmonella* LPS and then sacrificed at 36 hpt (*n* = 3) to analyze the impact of *Salmonella* LPS on chick T cell populations. **a** chT1+ cell ratios in spleen decreased but not significantly. **b**, **c** No significant change of CD4+/CD8+ T cells in chick thymus (**b**) or spleen (**c**) was observed. All data are presented as means ± SD. Statistically significant differences between LPS and saline groups at each time point were determined using Student’s *t*-test

In order to analyze changes in cell sub-populations during LPS stimulation, we detected chT1+, CD4+ and CD8+ T cells in the spleen and CD4+ and CD8+ T cells in the thymus at 36 h after LPS treatment using flow cytometry. The development of T cells in thymus undergoes stages of CD4-CD8- double negative (DN) cells, CD4 + CD8+ double positive (DP) cells and CD4 + CD8- or CD4-CD8+ single positive (SP) cells [1]. T cells migrated from thymus to the periphery express chT1 for a short time and chT1+ is typically used to assess recent thymic export [20–22]. Under LPS stimulation, the proportion of chT1+ cells in spleen tissue was not significantly different from that of the saline control group in this study. Proportions of DP and SP T cells also showed no significant difference in the spleen and thymus (Fig. 3). Therefore, both chT1 cell ratios in spleen and CD4+/CD8+ T cell ratios in thymus or spleen were not significantly changed at 36 h after LPS treatment.

### Identification of DETs in thymus after LPS challenge

**Fig. 4:**
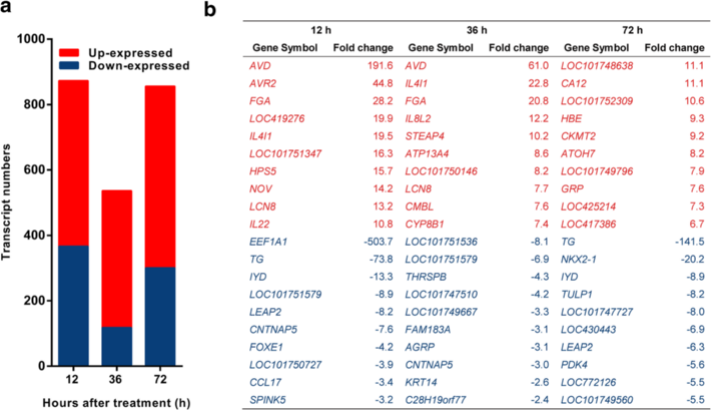
Analysis of DETs in chick thymus after *Salmonella* LPS treatment. Newly hatched chicks were injected i.p. with saline or 50 mg/kg *Salmonella* LPS and then sacrificed at defined time points (*n* = 7 ~ 9) to perform transcriptome analysis of the thymus. DETs were determined using the MARS model in DEGseq package between different time points (12 hpt vs. 0 hpt, 36 hpt vs. 0 hpt, 72 hpt vs. 0 hpt) with the same cut-off (*P* < 0.001, absolute fold change ≥ 1.5). **a** Numbers of DETs at different time point. **b** Top ten up- and down-expressed genes at different time point. The positive values indicate up-expression and negative values indicate down-expression

To determine the molecular mechanism of the effects induced by LPS treatment, we detected gene expression profiles in the thymus at 0, 12, 36 and 72 hpt using the RNA-seq method. In total, 76,769,194 clean reads were mapped to 27,391 transcripts. In order to identify the differential expressed transcripts (DETs), transcript expression levels at 12, 36 and 72 hpt were compared with those at 0 hpt. As a result, 873 DETs (507 up-expressed and 366 down-expressed) were identified at 12 hpt, 536 DETs (419 up-expressed and 117 down-expressed) at 36 hpt and 856 DETs (556 up-expressed and 300 down-expressed) at 72 hpt (Fig 4a; Additional file 2: Table S1) (absolute fold-change ≥ 1.5, *P* < 0.001). We found the maximum number of DETs at 12 hpt and the minimum number of DETs at 36 hpt. Many of the top 10 up- and down-expressed transcripts were related to immune responses, including *AVD*, *IL8L2*, *IL4I1* and *IL22* (Fig 4b; Additional file 2: Table S1).

### The expression patterns of ten genes in chick thymus were detected by qPCR

**Fig. 5:**
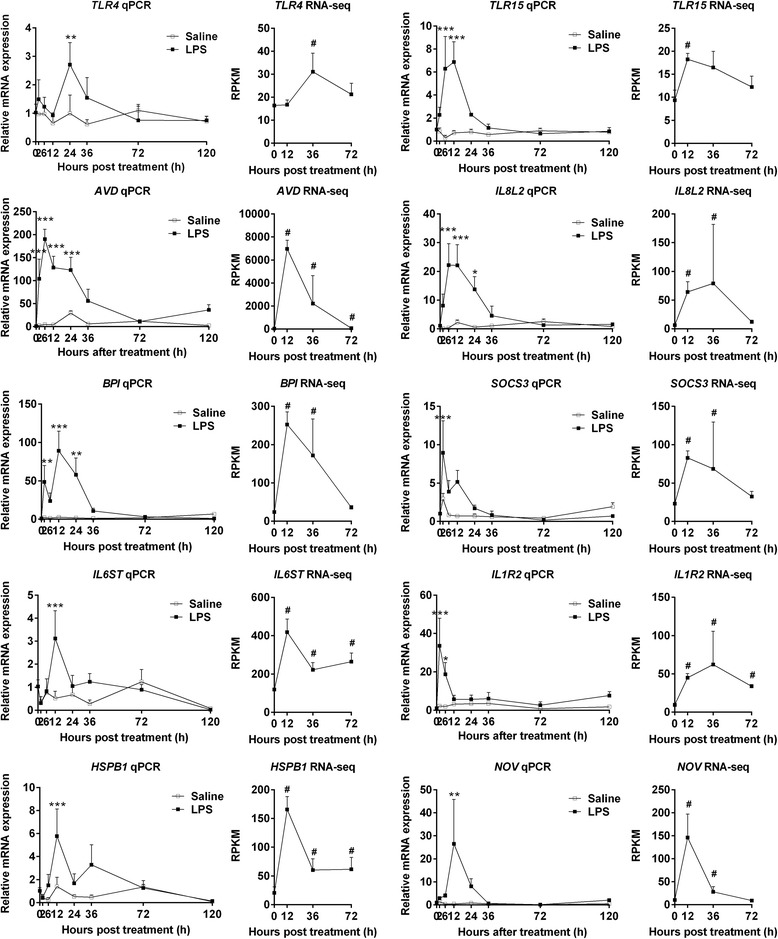
Confirmation of thymic DETs by qPCR. The qPCR analysis was conducted on ten genes (*TLR4*, *TLR15*, *AVD*, *IL8L2*, *BPI*, *SOCS3*, *IL6ST*, *IL1R2*, *HSPB1* and *NOV*) at 8 time points including 0, 2, 6, 12, 24, 36, 72 and 120 h after LPS challenge. Statistically significant differences for multiple comparisons (12 hpt vs. 0 hpt, 36 hpt vs. 0 hpt, 72 hpt vs. 0 hpt) were performed with Bonferroni’s multiple comparisons test after one-way ANOVA test. *P < 0.05, **P < 0.01, ***P < 0.001. RNA-seq was performed for the thymuses collected at 4 time points including 0, 12, 36, and 72 h after LPS challenge. The *P* values for transcript expression differences between different time points from RNA-seq were determined using MARS method. ^#^ *P* < 0.001. Results are presented as means ± SD (*n* = 3)

To validate the results of RNA-seq, ten up-expressed genes, including *TLR4*, *TLR15*, *AVD*, *IL8L2*, *BPI*, *SOCS3*, *IL6ST*, *IL1R2*, *HSPB1* and *NOV*, were chosen for qPCR detection. In order to accurately describe the expression patterns of genes, thymic tissue RNA at eight time points, including 0, 2, 6, 12, 24, 36, 72 and 120 h after LPS challenge, were collected for qPCR detection. The results showed that the gene expression patterns detected by qPCR were similar to those of obtained by the RNA-seq method (Fig. 5). There was a significant correlation between the changes of genes detected by RNA-seq and qPCR methods (Additional file 1: Figure S2). This finding confirmed the reliability of the RNA-seq data. Based on the qPCR results, all ten genes were up-expressed at 12 ~ 24 h after LPS treatment. Moreover, the *TLR15*, *AVD*, *IL8L2*, *BPI*, *SOCS3* and *IL1R2* genes were up-expressed as early as 2 h after LPS treatment, and most of them were restored to the normal level after 36 h of LPS treatment (Fig. 5). Similarly, all of these ten genes were up-expressed with STm infection. However, the peak of the gene expression under STm infection occurred later than that in the LPS-treated group (Additional file 1: Figure S3). This result indicated that the change in expression of genes during STm infection occurred later relative to that with LPS treatment. The difference in gene expression between LPS treatment and STm infection was consistent with the observed differences in thymocyte death and thymic atrophy between those two conditions. All of the results indicated that these biological events occurred later with STm infection than with LPS stimulation.

### TLR4-FOS/JUN signaling pathway may mediate LPS-induced thymic atrophy

**Fig. 6:**
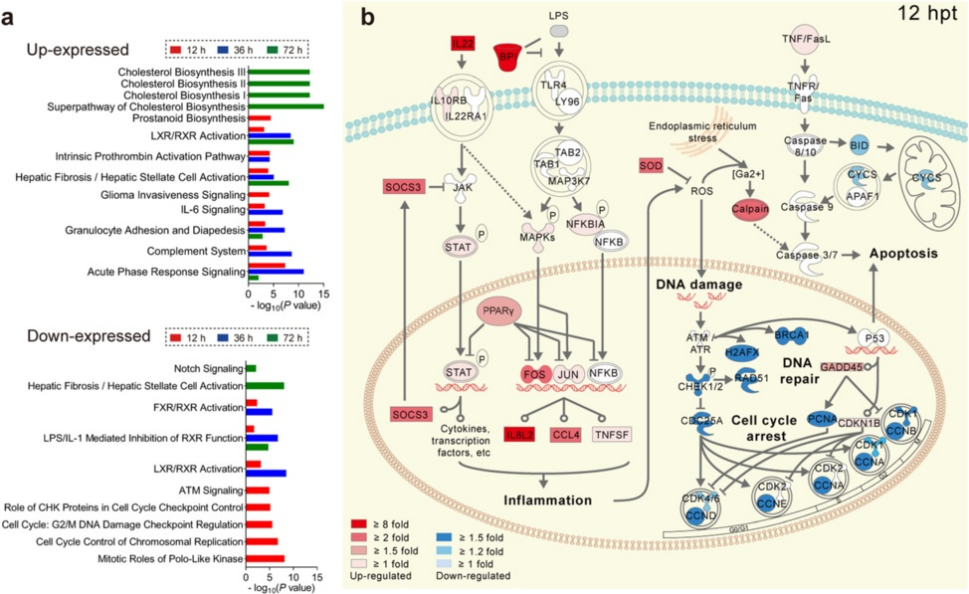
IPA pathway enrichment analysis of DETs in chick thymus after *Salmonella* LPS treatment. **a** Up-expressed or down-expressed DETs at each time point (12 hpt vs. 0 hpt, 36 hpt vs. 0 hpt, 72 hpt vs. 0 hpt) were subjected to IPA pathway enrichment analysis separately. The top five significant pathways enriched from up-expressed or down-expressed DETs at each time point are displayed separately. **b** Merged IPA pathways in the chick thymus at 12 hpt are illustrated based on enrichment analysis of IPA pathways. Red genes represent up-expressed genes, blue depict down-expressed genes and white symbols depict neighboring genes. The color intensity represents the average fold change

IPA was performed on DETs to identify the key signaling pathways triggered by LPS. We found that immune-related signaling pathways, including the complement system, granulocyte adhesion and diapedesis pathway, IL-6 signaling pathway and the acute phase response signaling pathway, were activated at 12 and 36 h post LPS treatment. Moreover, we found that cell cycle-related signaling pathways were down-regulated at 12 h post LPS treatment (Fig. 6a; Additional file 3: Table S2). We further analyzed the genes involved in the acute phase response signaling and cell cycle related pathways at 12 and 36 h. Genes, including *TLR4*, *FOS*, *JUN*, *IL22*, *IL8L2*, *SOCS3*, *PPARG*, were up-expressed in the TLR4-FOS/JUN signaling pathway. Genes, including *STT3A*, *CDC25A*, *CDK1/6*, *CCNA*, *CCNB*, *CCND* and *CCNE*, involved in the cell cycle pathway were down-expressed. Furthermore, the oxidative and calcium stress related genes of SOD and calpain were up-expressed (Fig. 6b; Additional file 1: Figure S5a; Additional file 4: Table S3). These changes were attenuated at 72 h after LPS treatment (Additional file 1: Figure S5b; Additional file 4: Table S3).

## Discussion

The thymus is a common target for infectious diseases without exception in mammals and birds [2, 4, 5]. In the present study, we found that STm induced acute thymic injury in newly hatched chicks, which was represented by thymocyte death and organic atrophy. Previous studies also found multiple pathogens, including parasites (e.g., *Ascaridia galli*), virus (e.g., chicken anemia agent) and bacteria (e.g., *E. coli*), could induce thymic atrophy in chickens [6, 9, 23]. Therefore, thymic atrophy is a common consequence of pathogenic infection in chickens.

We found both STm and *Salmonella* LPS could induce thymocyte death and thymic atrophy. They also induced almost the same patterns of gene expression, cell death and organic atrophy, although LPS acted more rapidly than STm. The maximum thymocyte death and thymic atrophy occurred at 12 and 36 h, respectively, with LPS challenge, while they occurred at 36 and 72 h, respectively, following STm infection. LPS has been confirmed to induce inflammation through the TLR4 signaling pathway [24–26]. Therefore, we deduced that LPS may be the major contributor to the thymus injury induced by STm infection, which acts by triggering intrathymic inflammation. Given the size of LPS versus bacteria, the delayed thymic reaction following STm infection may be resulted from the longer time for bacteria enter the tissue and interact with TLR4 than single LPS molecular. Besides, LPS treatment did not disrupt splenic chT1+ cells and CD4+/CD8+ T cell ratios in thymus and spleen tissues in chicks. The chT1 is an index to evaluate recent thymic export [21]. The maintenance of splenic chT1+ cells suggested that recent thymic export may not be disturbed at 36 h when maximum thymic atrophy occurred after LPS treatment. CD4 + CD8+ DP T cells are the main subpopulations in thymus [1]. The maintenance of DP and SP T cell ratios indicated that the composition of CD4+/CD8+ T cell subpopulations may not be changed at 36 h after LPS treatment. Our results were consistent with those of a previous study in mice infected with STm [13]. Thus, thymic dysfunction may not be inevitable with thymic injury due to pathogenic treatment.

To gain further insight into the acute chick thymic injury induced by *Salmonella* LPS, we performed transcriptome analysis of the thymus. We found that inflammatory cytokines, DNA damage genes and cell cycle genes were changed after LPS challenge. IPA results indicated that the acute inflammation mediated through the TLR4-FOS/JUN signaling pathway may play an important role in thymic atrophy. Based on the present studies and those findings published by other groups [24–39], we concluded that the transcriptional factors *FOS*/*JUN* were activated upon LPS binding to *TLR4* protein, consequently inducing the release of inflammatory factors *IL8L2* and *CCL4*. These inflammatory factors, including *IL-22*, *IL8L2* and *CCL4*, could induce the inflammation and promote the oxidative and calcium stress. These reactions resulted in DNA damage and cell cycle arrest, which contributed to the thymic atrophy in chicks. TLR4 is a specific pattern recognition receptor for LPS in birds and mammals [24, 27]. In mice, deficiency of TLR4 was shown to prevent thymocyte death and thymic atrophy when challenged by *E. coli* LPS [28, 29]. Transcriptional factors FOS/JUN can form the AP-1 dimeric complex to regulate cell proliferation and death [30]. AP-1 can be activated by LPS-TLR4 signaling and then induce the expression of inflammatory cytokines, including IL8 and CCL4, which contribute to inflammation by recruiting and activating leukocytes [27, 31, 32]. LPS stimulation also has been shown to induce the expression of another inflammatory factor IL22 *in vivo*, and overexpression of IL22 alone could induce thymic atrophy in mice [33, 34]. Furthermore, inflammatory reactions can promote the production of ROS [35, 36]. ROS alone can damage the host DNA, and it also can interact with calcium signaling to induce cell death and cell cycle arrest [37–39]. Thus, thymic atrophy induced by STm or *Salmonella* LPS in this study was considered to be mainly or at least partially mediated through the TLR4-FOS/JUN signaling pathway in chicks.

We also found that some anti-inflammatory factors, including *BPI*, *PPARγ* and *SOCS3*, were activated after LPS stimulation. BPI can inhibit formation of the LPS-TLR4 complex through competing with LPS [40]. PPARγ can inhibit FOS/JUN and NF-κB by preventing their binding to target sequences [41, 42]. SOCS3 can inhibit STAT phosphorylation through binding of JAK kinase [43]. Thus, negative inflammation response regulators naturally can also be activated when the host is infected by pathogens in order to maintain the immune homeostasis and avoid excessive tissue injury [44, 45]. In addition, we observed apoptosis mediated through the mitochondrial pathway at 72 h of LPS treatment. Moreover, the thymus weight was restored to the normal level at 120 h. These finding indicated that compensatory growth occurred after the acute inflammatory injury. The phenomenon of compensatory growth of the thymus after injury also has been observed in mice [46].

## Conclusions

We confirmed that STm and *Salmonella* LPS could induce thymocyte death and thymic atrophy in chicks. LPS could be the main factor in the thymic atrophy during a STm infection. Inflammatory reactions were the factor contributing to the thymic atrophy after LPS treatment in chicks. Moreover, this work associated with findings published by other groups [24–39] demonstrates that this inflammatory process may be mediated through the TLR4-FOS/JUN signaling pathway. Our results provide novel evidence for the molecular mechanism of the thymus injury induced by LPS derived from STm.

## Methods

### Ethics statement

This study was approved by the Ethics Committee of Huazhong Agricultural University (HZAUMU2013-0005). The experiments were performed in accordance with the Guide for the Care and Use of Laboratory Animals (1996), and protocols were approved by the Hubei Province for Biological Studies Animal Care and Use Committee.

### Animals

Healthy newly hatched broiler chicks (≤ 3 days old, Cobb 500) were obtained from the Wuhan Zhengda chicken breeding company and housed under conventional conditions without any vaccinations. Chicks were injected intraperitoneally (i.p.) with 0.5 mL of 5 × 10^4^ CFU/mL of live STm strain cvcc541 (China Veterinary Culture Collection Center, Beijing, China) or LPS derived from STm (L7261; Sigma-Aldrich, St. Louis, MO, USA) at 50 mg/kg of body weight in 0.5 mL avian saline (0.75 % NaCl). The control group was injected only with 0.5 mL avian saline. Six chicks per treatment (STm or saline injection) per time point (0, 12, 36, 72 and 120 h) were sampled in STm infection experiment. At least four chicks per treatment (LPS or saline injection) per time point (0, 2, 6, 12, 24, 36, 72 and 120 h) were sampled for morphology and qPCR analyses. For transcriptome analysis of chick thymus after LPS treatment, at least seven chicks per time point (0, 12, 36 and 72 h) were sampled. Thymuses were collected and weighed at defined time points. The thymus index was calculated using the formula: Thymus index (mg/g) = weight of thymus (mg)/body weight (g) [47]. For morphological analysis, fresh thymuses were fixed in 4 % paraformaldehyde, embedded into paraffin and stored at 4 °C. For gene expression analysis, fresh thymuses were frozen in liquid nitrogen and stored at -70 °C. For cell subpopulation analysis, fresh thymuses and spleens were collected and performed flow cytometric assays without storing. All organ sampling for each chick was finished in thirty minutes.

### Hematoxylin and eosin staining

Thymic tissues embedded in paraffin were cut into 4-μm-thick sections. The sections were then deparaffinized in xylene, rehydrated in a graded series of alcohol to water. Slides were stained with Harris hematoxylin solution for 5 min and differentiated in acid alcohol for 10 s. After rinsing in running tap water for 15 min, the sections were then stained in eosin solution for 1 min, dehydrated in alcohol, cleared in xylene and mounted with coverslips. Stained samples were examined by light microscopy (Olympus BX51, Tokyo, Japan) with a digital camera (DP72; Olympus).

### Formamide-monoclonal antibody (MAb) assay

The rehydrated thymic sections were incubated with 20 μg/ml proteinase K and 0.1 mg/ml saponin in PBS for 20 min at room temperature (RT) and then incubated in 50 % (v/v) formamide in distilled water at 56 °C for 20 min before transferring to cold PBS for 5 min. The sections were then incubated with a primary mouse IgM anti-ssDNA MAb (clone F7-26; EMD Millipore, Billerica, MA, USA) at a dilution of 1:10 for 30 min at 37 °C. Secondary antibody incubation was performed using the anti-mouse IgM SABC kit (Boster, Wuhan, China). After visualization with DAB solution, the sections were counterstained with hematoxylin. Sections were examined by light microscopy (Olympus BX51) with a digital camera (DP72; Olympus). Sections were sampled by random movement of the mechanical microscope stage to bring new, non-overlapping areas (at least 5 fields) into view. Counts were standardized from the mean number of formamide-MAb-positive cells per field to the number per 1 × 10^5^ μm^2^.

### TUNEL assay

The TUNEL assay was performed using the In Situ Cell Death Detection Kit, POD (Roche Boehringer Mannheim Corp., Indianapolis, IN, USA) according to the manufacturer’s instruction. The rehydrated thymic sections were incubated with terminal deoxynucleotidyl transferase (TdT) in a reaction buffer for 1 h at 37 °C. Thereafter, the sections were washed in PBS and observed using a fluorescence microscope (Olympus, Tokyo, Japan) with a digital camera (DP72; Olympus) before the following steps were performed. Endogenous peroxides were blocked by incubation in 3 % H_2_O_2_ in PBS for 10 min at RT. Fluorescent signal conversion was performed using an anti-fluorescein antibody conjugated with peroxides. Sections were incubated with DAB solution and counterstained with hematoxylin. Cell counting and statistical analysis for TUNEL-positive cells were conducted in the same manner as that for formamide-MAb-positive cells mentioned above.

### Flow cytometry

Thymus and spleen tissues were prepared to obtain single-cell suspensions (2 × 10^6^ cells) as previously described [48]. Immunofluorescence staining was performed with anti-chicken directly-conjugated MAbs: anti-CD4 FITC, anti-CD8 PE and anti-chT1 FITC (AbD Serotec, Ltd., Oxford, UK). Cell suspensions were added to PBS wash buffer containing 2 % bovine serum albumin and diluted antibodies for 30 min at 4 °C, followed by washing and resuspension in PBS containing 1 % paraformaldehyde. All cell populations were assessed using a FACSAria III (BD Biosciences, San Jose, CA, USA).

### RNA-seq and data analysis

Total RNA was isolated from each thymic sample using the standard TRIzol protocol (Invitrogen, Carlsbad, CA, USA). RNA quality was examined by gel electrophoresis and with a Nanodrop spectrophotometer (Thermo, Waltham, MA, USA). For RNA sequencing, RNA samples from seven to nine biological replicates at each time point (0, 12, 36 and 72 h) were separated into three independent pools, each comprised of two or three distinct samples, at equal amounts. Strand-specific libraries were constructed using the TruSeq RNA sample preparation kit (Illumina, San Diego, CA, USA), and sequencing was carried out using the Illumina HiSeq 2000 instrument by the commercial service of Genergy Biotechnology Co. Ltd. (Shanghai, China). The raw data was handled by Perl and data quality was checked by FastQC 0.11.2 (http://www.bioinformatics.babraham.ac.uk/projects/fastqc/). The read length was 50 bp. Clean reads were aligned to the chicken genome (release: *Gallus gallus* 4.0) from NCBI using Bowtie [49], with one mismatch allowed. Genome mapped data were annotated using the gff3 file of the *Gallus gallus* 4.0 genome (ftp://ftp.ncbi.nlm.nih.gov/genomes/Gallus_gallus/). The expression of the transcript was calculated by RPKM (Reads Per Kilobase of exon model per Million mapped reads) using Perl. Differentially expression transcripts (DETs) were determined using the MA-plot-based method with Random Sampling (MARS) model in the DEGseq package between different time points (12 hpt vs. 0 hpt, 36 hpt vs. 0 hpt, 72 hpt vs. 0 hpt) [50]. Generally, in MARS model, *M* = log_2_*C*_*1*_ - log_2_*C*_*2*_, and *A* = (log_2_*C*_*1*_ + log_2_*C*_*2*_)/2 (*C*_*1*_ and *C*_*2*_ denote the counts of reads mapped to a specific gene obtained from two samples) [50]. The thresholds for determining DETs are *P* < 0.001 and absolute fold change ≥ 1.5. Then DETs were chosen for signaling pathway enrichment analysis using Ingenuity Pathway Analysis (IPA) software. The significantly enriched pathways were determined when *P* < 0.05 and at least two affiliated genes were included.

### Quantitative real-time PCR (qPCR)

Total RNA was isolated from thymuses of three chicks at each time point (0, 2, 6, 12, 24, 36, 72 and 120 h) of LPS treatment group or saline control group. In addition, RNA was also isolated from thymuses of three chicks at each time point (0, 12, 36, 72 and 120 h) under STm infection. Then total RNA were treated with RNase-free DNase I (Fermentas, Opelstrasse, Germany) to remove contaminating genomic DNA. The first strand cDNA was synthesized using the RevertAid First Strand cDNA Synthesis Kit (Fermentas, Opelstrasse, Germany). The reaction mixture (10 μl) for qPCR contained of 5 μL SYBR Select Master Mix for CFX (Applied Biosystems), 0.2 μL of each forward and reverse primer and 1 μL of template cDNA. The qPCR reactions were performed on a Bio-Rad CFX Connect real-time PCR detection system (Bio-Rad, Hercules, CA, USA). The qPCR conditions were as follows: pre-denaturation at 95 °C for 5 min, followed by 40 cycles of denaturation at 95 °C for 30 s, annealing at 60 °C for 30 s, and elongation at 72 °C for 20 s. The primer sequences were listed in Additional file 5: Table S4. ACTB was chosen as a reference for qPCR. All samples were run in triplicate and gene expression levels were quantified using the ΔΔCt method [51].

### Statistical analysis

Data were calculated as the means ± standard deviation (SD). All analyses and graphic representations were performed with Prism software 5.01 (GraphPad Software, Inc., San Diego, USA). The statistical significance in mean values between two-group comparison was performed using two-tailed Student’s *t*-test (Fig. 1a and b; Fig. 2; Fig. 3; Additional file 1: Figure S1). The statistical significance in the comparison of multiple sample sets versus control was performed with Bonferroni’s multiple comparisons test after one-way ANOVA test (Fig. 1c and d; Fig. 5). The correlation analysis between changes of ten genes detected by qPCR and RNA-seq was performed and the significance was detected using Pearson’s test (Additional file 1: Figure S2). Differences were considered significant if *P* < 0.05. *P < 0.05, **P < 0.01 and ***P < 0.001.

### Availability of supporting data

All the datasets supporting the results have been listed in the article and its additional files. The RNA-seq data has been deposited to the National Center for Biotechnology Information (NCBI) Short Read Archive (SRA) under accession code SRP065372.

## Additional files

Additional file 1: Supplementary figures. (PDF 1259 kb) Additional file 2: Table S1. Detailed information on DETs at different time points. a Up-expressed DETs at 12 hpt. b Down-expressed DETs at 12 hpt. c Up-expressed DETs at 36 hpt. d Down-expressed DETs at 36 hpt. e Up-expressed DETs at 72 hpt. f Down-expressed DETs at 72 hpt. Transcripts were considered as DETs only with absolute fold changes ≥ 1.5 and *P* < 0.001. (XLS 387 kb) Additional file 3: Table S2. Detailed information on IPA pathway analysis of DETs at different time points. a Over-represented pathways from up-expressed DETs at 12 hpt. b Over-represented pathways from down-expressed DETs at 12 hpt. c Over-represented pathways from up-expressed DETs at 36 hpt. d Over-represented pathways from down-expressed DETs at 36 hpt. e Over-represented pathways from up-expressed DETs at 72 hpt. f Over-represented pathways from down-expressed DETs. IPA pathways with *P* < 0.05 and at least two genes affiliated were reported in the present study. (XLS 63 kb) Additional file 4: Table S3. Detailed information on genes involved in merged IPA pathways at different time point. Genes involved in merged IPA pathways with *P* < 0.001 were considered. Dashes indicated no significant difference in the genes expression. (XLS 21 kb) Additional file 5: Table S4. List of qPCR primers. (XLS 27 kb)

## References

[1] Gameiro J, Nagib P, Verinaud L (2010). The thymus microenvironment in regulating thymocyte differentiation. Cell Adh Migr.

[2] Raviola E, Karnovsky MJ (1972). Evidence for a blood-thymus barrier using electron-opaque tracers. J Exp Med.

[3] Savino W (2006). The thymus is a common target organ in infectious diseases. PLoS Pathog.

[4] Nunes-Alves C, Nobrega C, Behar SM, Correia-Neves M (2013). Tolerance has its limits: how the thymus copes with infection. Trends Immunol.

[5] Hoerr FJ (2010). Clinical aspects of immunosuppression in poultry. Avian Dis.

[6] Nakamura K, Imada Y, Maeda M (1986). Lymphocytic depletion of bursa of Fabricius and thymus in chickens inoculated with Escherichia coli. Vet Pathol.

[7] Nakamura K, Yuasa N, Abe H, Narita M (1990). Effect of infectious bursal disease virus on infections produced by Escherichia coli of high and low virulence in chickens. Avian Pathol.

[8] Ramadan HH, Abou Znada NY (1991). Some pathological and biochemical studies on experimental ascaridiasis in chickens. Nahrung.

[9] Sadun EH (1950). Studies on the pathogenicity in chickens of single infections of variable size with the Nematode, Ascaridia Galli. Poult Sci.

[10] Newell DG, Koopmans M, Verhoef L, Duizer E, Aidara-Kane A, Sprong H, Opsteegh M, Langelaar M, Threfall J, Scheutz F (2010). Food-borne diseases - the challenges of 20 years ago still persist while new ones continue to emerge. Int J Food Microbiol.

[11] Barrow PA (2000). The paratyphoid salmonellae. Rev Sci Tech.

[12] Deobagkar-Lele M, Victor ES, Nandi D (2014). c-Jun NH2 -terminal kinase is a critical node in the death of CD4+ CD8+ thymocytes during Salmonella enterica serovar Typhimurium infection. Eur J Immunol.

[13] Ross EA, Coughlan RE, Flores-Langarica A, Lax S, Nicholson J, Desanti GE, Marshall JL, Bobat S, Hitchcock J, White A (2012). Thymic function is maintained during Salmonella-induced atrophy and recovery. J Immunol.

[14] Withanage GS, Kaiser P, Wigley P, Powers C, Mastroeni P, Brooks H, Barrow P, Smith A, Maskell D, McConnell I (2004). Rapid expression of chemokines and proinflammatory cytokines in newly hatched chickens infected with Salmonella enterica serovar typhimurium. Infect Immun.

[15] Withanage GS, Wigley P, Kaiser P, Mastroeni P, Brooks H, Powers C, Beal R, Barrow P, Maskell D, McConnell I (2005). Cytokine and chemokine responses associated with clearance of a primary Salmonella enterica serovar Typhimurium infection in the chicken and in protective immunity to rechallenge. Infect Immun.

[16] Martin SJ, Green DR (1995). Protease activation during apoptosis: death by a thousand cuts?. Cell.

[17] Frankfurt OS, Robb JA, Sugarbaker EV, Villa L (1996). Monoclonal antibody to single-stranded DNA is a specific and sensitive cellular marker of apoptosis. Exp Cell Res.

[18] Frankfurt OS, Robb JA, Sugarbaker EV, Villa L (1997). Apoptosis in breast carcinomas detected with monoclonal antibody to single-stranded DNA: relation to bcl-2 expression, hormone receptors, and lymph node metastases. Clin Cancer Res.

[19] Grasl-Kraupp B, Ruttkay-Nedecky B, Koudelka H, Bukowska K, Bursch W, Schulte-Hermann R (1995). In situ detection of fragmented DNA (TUNEL assay) fails to discriminate among apoptosis, necrosis, and autolytic cell death: a cautionary note. Hepatology.

[20] Katevuo K, Imhof BA, Boyd R, Chidgey A, Bean A, Dunon D, Gobel TW, Vainio O (1999). ChT1, an Ig superfamily molecule required for T cell differentiation. J Immunol.

[21] Kong F, Chen CH, Cooper MD (1998). Thymic function can be accurately monitored by the level of recent T cell emigrants in the circulation. Immunity.

[22] Kong FK, Chen CL, Six A, Hockett RD, Cooper MD (1999). T cell receptor gene deletion circles identify recent thymic emigrants in the peripheral T cell pool. Proc Natl Acad Sci U S A.

[23] Jeurissen SH, Pol JM, de Boer GF (1989). Transient depletion of cortical thymocytes induced by chicken anaemia agent. Thymus.

[24] Brownlie R, Allan B (2011). Avian toll-like receptors. Cell Tissue Res.

[25] Lu YC, Yeh WC, Ohashi PS (2008). LPS/TLR4 signal transduction pathway. Cytokine.

[26] Hoshino K, Takeuchi O, Kawai T, Sanjo H, Ogawa T, Takeda Y, Takeda K, Akira S (1999). Cutting edge: Toll-like receptor 4 (TLR4)-deficient mice are hyporesponsive to lipopolysaccharide: evidence for TLR4 as the Lps gene product. J Immunol.

[27] Akira S, Takeda K (2004). Toll-like receptor signalling. Nat Rev Immunol.

[28] Wang SD, Huang KJ, Lin YS, Lei HY (1994). Sepsis-induced apoptosis of the thymocytes in mice. J Immunol.

[29] Norimatsu M, Ono T, Aoki A, Ohishi K, Tamura Y (1995). In-vivo induction of apoptosis in murine lymphocytes by bacterial lipopolysaccharides. J Med Microbiol.

[30] Shaulian E, Karin M (2002). AP-1 as a regulator of cell life and death. Nat Cell Biol.

[31] Li X, Jiang S, Tapping RI (2010). Toll-like receptor signaling in cell proliferation and survival. Cytokine.

[32] Ono SJ, Nakamura T, Miyazaki D, Ohbayashi M, Dawson M, Toda M (2003). Chemokines: roles in leukocyte development, trafficking, and effector function. J Allergy Clin Immunol.

[33] Liang SC, Nickerson-Nutter C, Pittman DD, Carrier Y, Goodwin DG, Shields KM, Lambert AJ, Schelling SH, Medley QG, Ma HL (2010). IL-22 induces an acute-phase response. J Immunol.

[34] Weber GF, Schlautkotter S, Kaiser-Moore S, Altmayr F, Holzmann B, Weighardt H (2007). Inhibition of interleukin-22 attenuates bacterial load and organ failure during acute polymicrobial sepsis. Infect Immun.

[35] Mittal M, Siddiqui MR, Tran K, Reddy SP, Malik AB (2014). Reactive oxygen species in inflammation and tissue injury. Antioxid Redox Signal.

[36] Hakim J (1993). Reactive oxygen species and inflammation. C R Seances Soc Biol Fil.

[37] Forman HJ, Torres M (2002). Reactive oxygen species and cell signaling: respiratory burst in macrophage signaling. Am J Respir Crit Care Med.

[38] Nathan C, Cunningham-Bussel A (2013). Beyond oxidative stress: an immunologist’s guide to reactive oxygen species. Nat Rev Immunol.

[39] Harada A, Sekido N, Akahoshi T, Wada T, Mukaida N, Matsushima K (1994). Essential involvement of interleukin-8 (IL-8) in acute inflammation. J Leukoc Biol.

[40] Weiss J (2003). Bactericidal/permeability-increasing protein (BPI) and lipopolysaccharide-binding protein (LBP): structure, function and regulation in host defence against Gram-negative bacteria. Biochem Soc Trans.

[41] Clark RB (2002). The role of PPARs in inflammation and immunity. J Leukoc Biol.

[42] Chinetti G, Fruchart JC, Staels B (2000). Peroxisome proliferator-activated receptors (PPARs): nuclear receptors at the crossroads between lipid metabolism and inflammation. Inflamm Res.

[43] Carow B, Rottenberg ME (2014). SOCS3, a major regulator of infection and inflammation. Front Immunol.

[44] Yoshimura A, Suzuki M, Sakaguchi R, Hanada T, Yasukawa H (2012). SOCS, inflammation, and autoimmunity. Front Immunol.

[45] Matarese G, La Cava A (2004). The intricate interface between immune system and metabolism. Trends Immunol.

[46] Hick RW, Gruver AL, Ventevogel MS, Haynes BF, Sempowski GD (2006). Leptin selectively augments thymopoiesis in leptin deficiency and lipopolysaccharide-induced thymic atrophy. J Immunol.

[47] Sharma JM, Dohms JE, Metz AL (1989). Comparative pathogenesis of serotype 1 and variant serotype 1 isolates of infectious bursal disease virus and their effect on humoral and cellular immune competence of specific-pathogen-free chickens. Avian Dis.

[48] Billard MJ, Gruver AL, Sempowski GD (2011). Acute endotoxin-induced thymic atrophy is characterized by intrathymic inflammatory and wound healing responses. PLoS One.

[49] Langmead B, Trapnell C, Pop M, Salzberg SL (2009). Ultrafast and memory-efficient alignment of short DNA sequences to the human genome. Genome Biol.

[50] Wang L, Feng Z, Wang X, Wang X, Zhang X (2010). DEGseq: an R package for identifying differentially expressed genes from RNA-seq data. Bioinformatics.

[51] Livak KJ, Schmittgen TD (2001). Analysis of relative gene expression data using real-time quantitative PCR and the 2(-Delta Delta C(T)) Method. Methods.

