# Supplementary material for: Transcriptome analysis indicated that Salmonella lipopolysaccharide-induced thymocyte death and thymic atrophy were related to TLR4-FOS/JUN pathway in chicks
Source: BMC Genomics. 2016 May 4;17:322. doi: 10.1186/s12864-016-2674-6 (PMC4855877; doi:10.1186/s12864-016-2674-6)
Supplement: Additional file 1: Supplementary figures. — (PDF 1259 kb) [file 12864_2016_2674_MOESM1_ESM.pdf]

Transcriptome analysis indicated that *Salmonella* lipopolysaccharide-induced thymocyte death and thymic atrophy were related to TLR4-FOS/JUN pathway in chicks

Haibo Huang, An Liu, Hui Wu, Abdur Rahman Ansari, Jixiang Wang, Xiyao Huang, Xing Zhao, Kemei Peng, Juming Zhong and Huazhen Liu

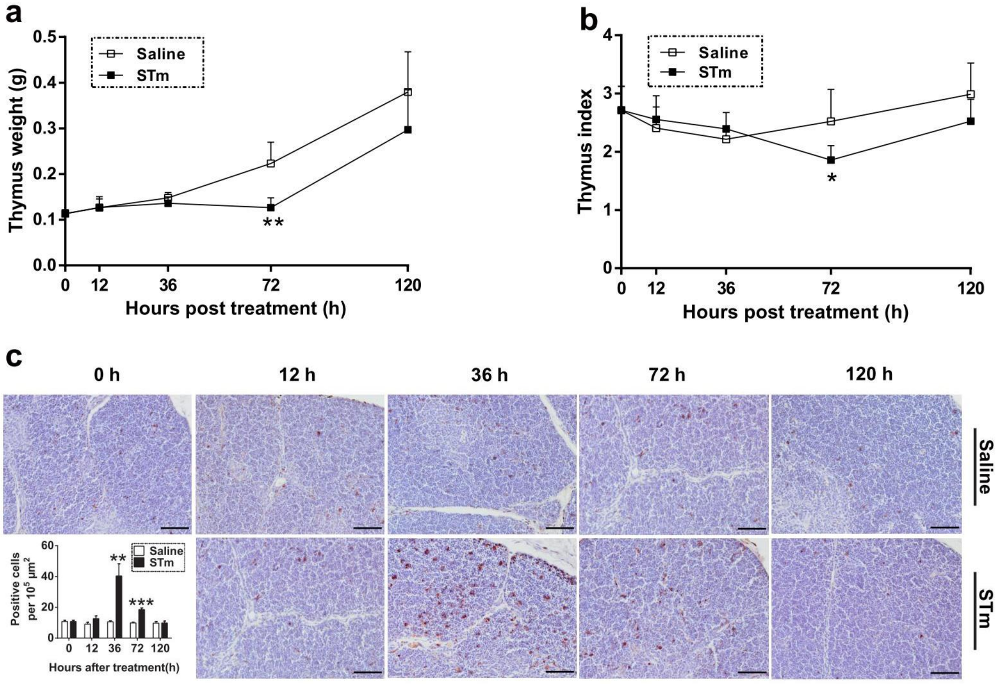

Figure. S1 STm induced acute thymic injury in chicks. Newly hatched chicks were injected i.p. with 0.5 mL 75% saline or 5×10^4 CFU/mL STm and sacrificed at defined time points to evaluate thymic injury. **a**, **b** STm infection reduced thymus weight and index at 72 hpt in chicks (n = 6). **c** STm infection increased thymocyte death at 36 and 72 hpt in chicks (n = 4~5). The positive cells were mainly distributed in thymic cortex. At least 5 fields in each section of the thymus were sampled, and positive cells per 1×10^5 μm^2 in the thymic cortex were quantified. Scare bars = 50 μm. All data are presented as means ± SD. Statistically significant differences between STm and saline groups at each time point were determined using Student's t-test. *$P < 0.05$, **$P < 0.01$, ***$P < 0.001$.

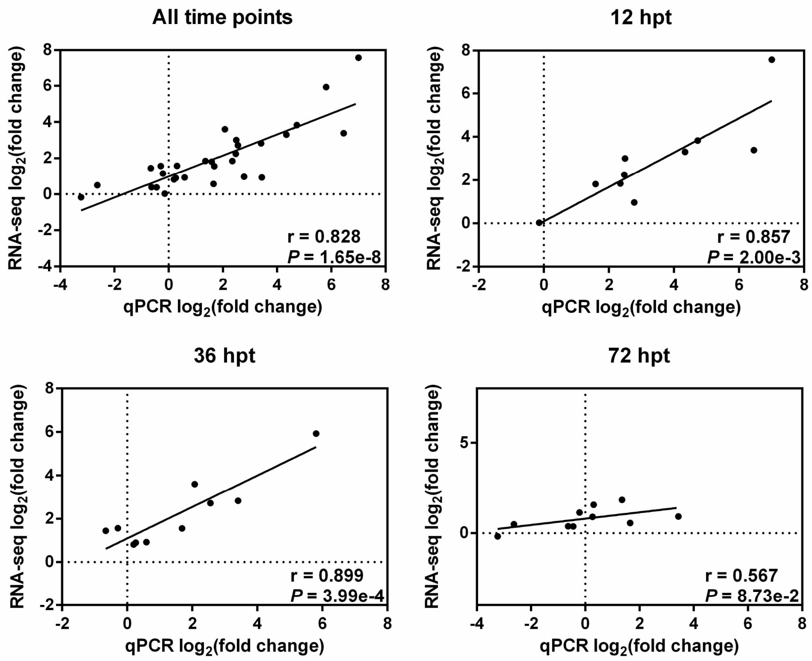

Figure. S2 Correlation analysis between changes of ten genes (*TLR4, TLR15, AVD, IL8L2*, *BPI*, *SOCS3*, *IL6ST*, *IL1R2*, *HSPB1* and *NOV*) detected by RNA-seq and qPCR methods. Newly hatched chicks were injected i.p. with 50 mg/kg *Salmonella* LPS and sacrificed at defined time points. The $\log_2$(fold change) values were calculated from relative mRNA expression values (for qPCR data) or RPKM values (for RNA-seq data) between different time points (12 hpt vs. 0 hpt, 36 hpt vs. 0 hpt, 72 hpt vs. 0 hpt). The significance of correlation analysis was detected using Pearson's test. Each point represents one gene per comparisons.

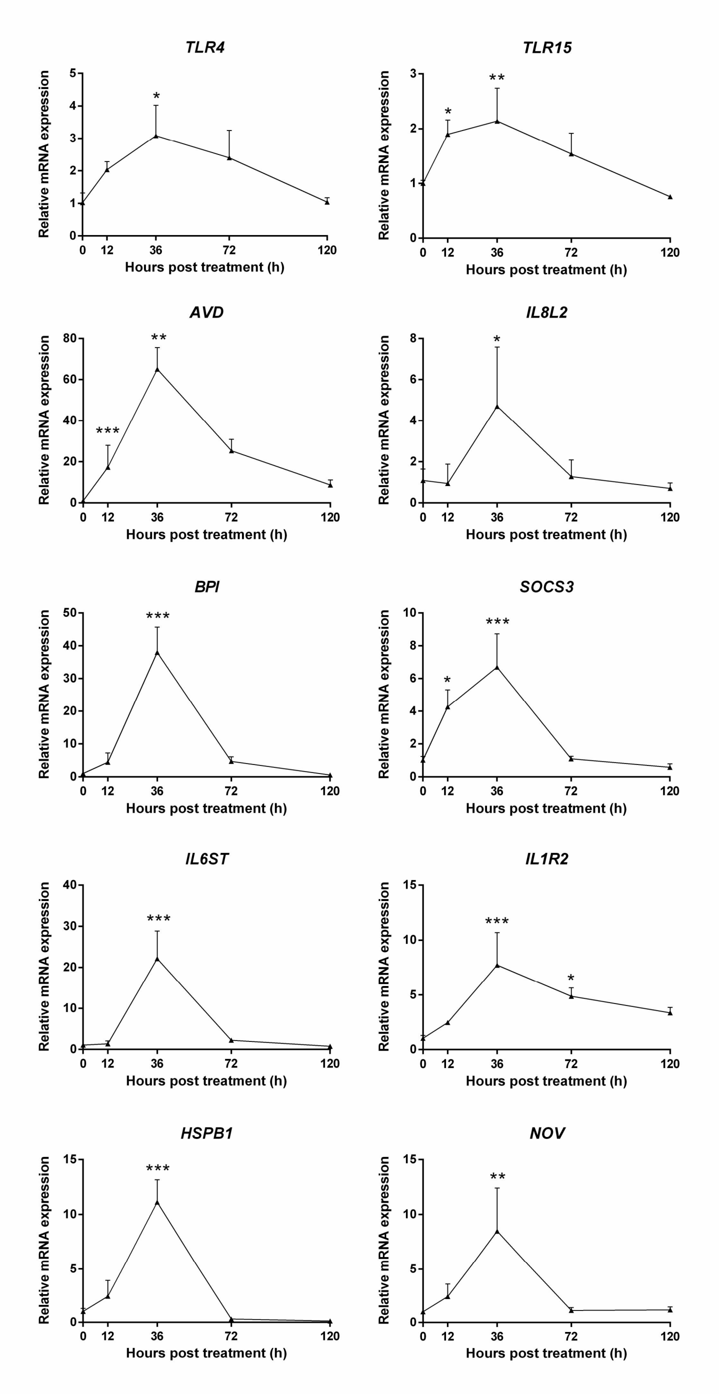

Figure. S3 Expression of ten genes in chick thymus after STm infection. The qPCR was conducted on ten genes (*TLR4, TLR15, AVD, IL8L2, BPI, SOCS3, IL6ST, IL1R2, HSPB1* and *NOV*) in chicks (n = 3) after STm infection. Statistically significant differences for multiple comparisons (12 hpt vs. 0 hpt, 36 hpt vs. 0 hpt, 72 hpt vs. 0 hpt) were performed with Bonferroni's multiple comparisons test after one-way ANOVA test. *P < 0.05, **P < 0.01, ***P < 0.001.

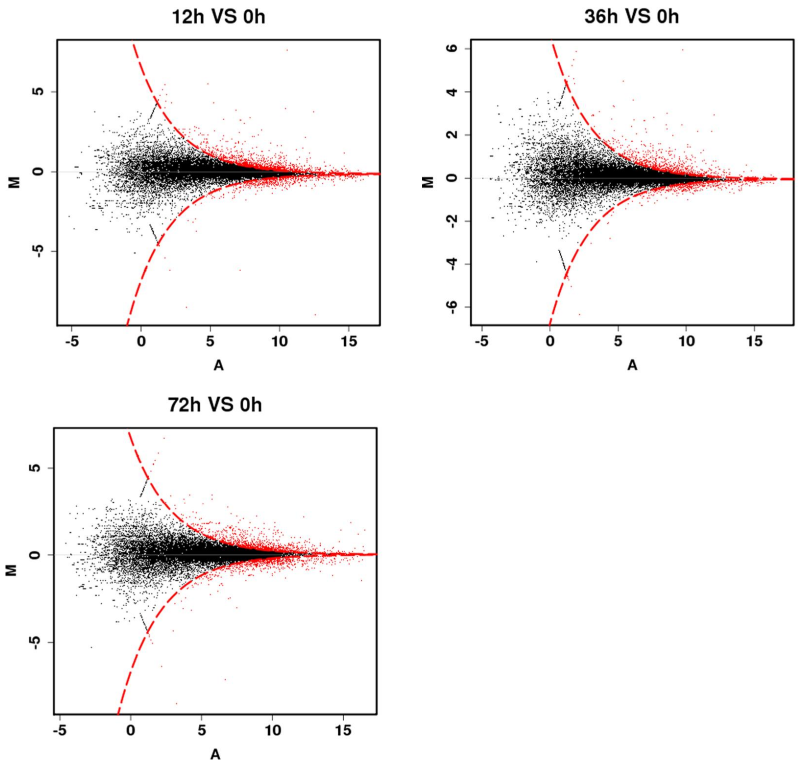

Figure. S4 Visualization of differential expression from RNA-Seq data using MA-plots. Differential gene expression was depicted as MA-plots between different time points (12 hpt vs. 0 hpt, 36 hpt vs. 0 hpt, 72 hpt vs. 0 hpt). The red line indicate significance threshold ($P < 0.001$). Red points represent transcripts with $P < 0.001$.

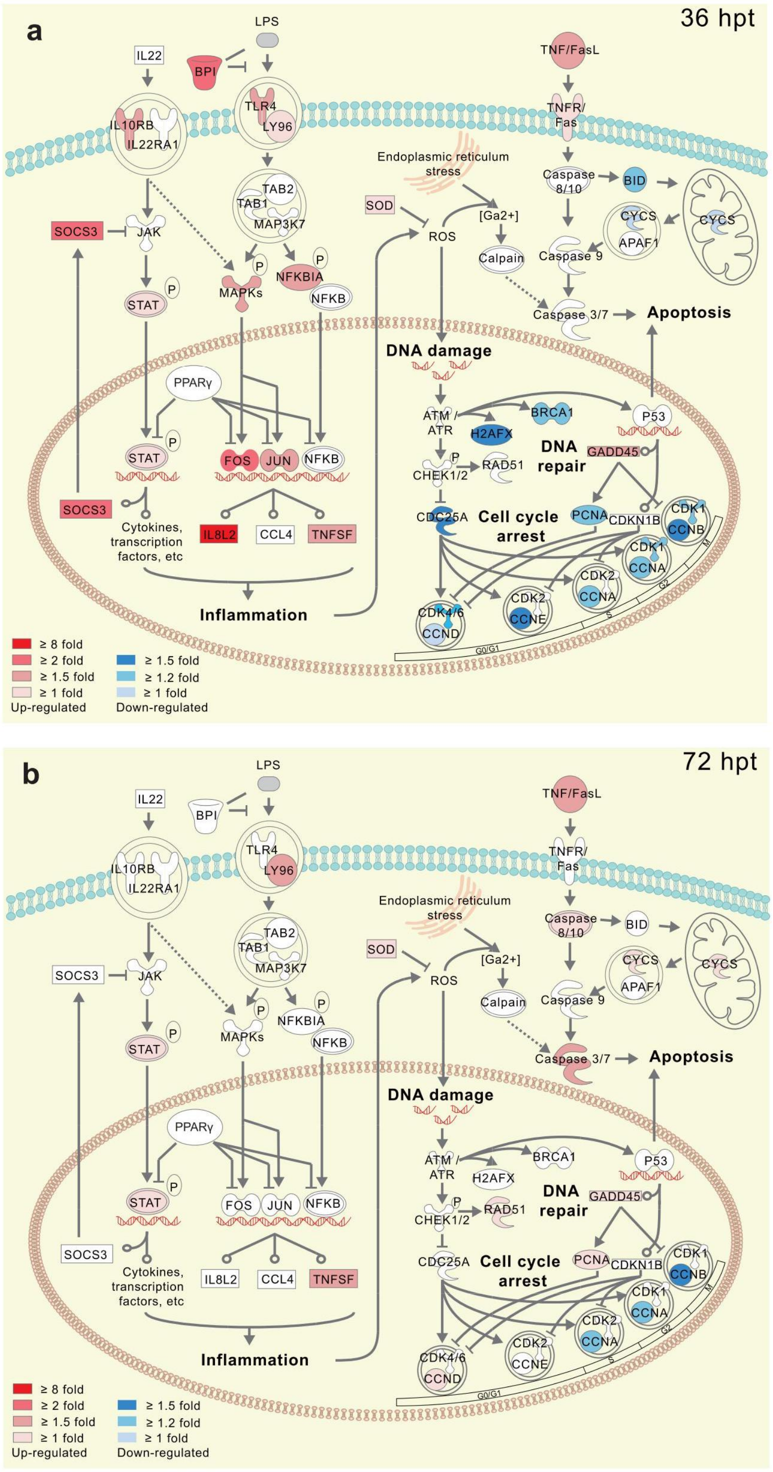

Figure. S5 IPA pathway enrichment analysis of DEGs in chick thymus at 36 and 72 hpt after *Salmonella* LPS treatment. Merged IPA pathways in chick thymus at 36 hpt (**a**) and 72 hpt (**b**) are illustrated based on enrichment analysis of IPA pathways. Red genes represent up-expressed genes, blue depict down-expressed genes and white symbols depict neighboring genes. The color intensity represents the average fold change.
